# Crystal structure of a two-dimensional coordination polymer of formula [Zn(NDC)(DEF)] (H_2_NDC is naphthalene-2,6-di­carb­oxy­lic acid and DEF is *N*,*N*-di­ethyl­formamide)

**DOI:** 10.1107/S2056989019014142

**Published:** 2019-10-29

**Authors:** Nathalie Saffon-Merceron, Alain Vigroux, Pascal Hoffmann

**Affiliations:** a Université de Toulouse, UPS, Institut de Chimie de Toulouse, ICT FR 2599, 118 route de Narbonne, F-31062 Toulouse, France

**Keywords:** crystal structure, metal–organic framework, naphthalenedi­carb­oxy­lic acid, *N*,*N*-di­ethyl­formamide, zinc carboxyl­ates

## Abstract

A zinc metal–organic framework, namely poly[bis­(*N*,*N*-di­ethyl­formamide)(μ_4_-naphthalene-2,6-di­carboxyl­ato)(μ_2_-naphthalene-2,6-di­carboxyl­ato)dizinc(II)], [Zn(C_12_H_6_O_4_)(C_15_H_11_NO)]_*n*_, built from windmill-type secondary building units and forming zigzag shaped two-dimensional stacked layers, has been solvothermally synthesized from naphthalene-2,6-di­carb­oxy­lic acid and zinc(II) acetate as the metal source in *N*,*N*-di­ethyl­formamide containing small amounts of formic acid.

## Abstract 2

The zinc metal organic framework poly[bis­(*N*,*N*-di­ethyl­formamide)(μ_4_-naphthalene-2,6-di­carboxyl­ato)(μ_2_-naphthalene-2,6-di­carboxyl­ato)dizinc(II)], built from windmill-type secondary building units and forming zigzag shaped two-dimensional stacked layers, has been solvothermally synthesized from naphthalene-2,6-di­carb­oxy­lic acid and zinc(II) acetate as the metal source in *N*,*N*-di­ethyl­formamide containing small amounts of formic acid.

## Chemical context   

In a preceding study, we showed how the presence of a small amount of added organic acids in the solvent *N*,*N*-di­ethyl­formamide (DEF), under solvothermal conditions, can be crucial in steering the production of new MOF (metal–organic framework) structures, as exemplified by the formation of two new zinc–terephthalate MOFs based on the trinuclear Zn_3_(O_2_C*R*)_6_ secondary building unit (SBU) and containing the formate anion, solvothermally obtained from the well-studied MOF-5 system Zn/H_2_BDC/DEF (H_2_BDC = benzene-1,4-dicarboxylic acid) in the presence of small amounts of added formic acid (Saffon-Merceron *et al.*, 2015 ▸). Here, another ligand, NDC^2−^ (H_2_NDC = naphthalene-2,6-di­carb­oxy­lic acid) is considered to further study the influence of added formic acid in DEF in MOF synthesis. The NDC^2−^ ligand has been widely used previously to prepare a number of MOFs (Gangu *et al.*, 2017 ▸), including IRMOF-8 belonging to the isoreticular MOF series IRMOF-1-16, which have the same underlying topology as MOF-5 with oxygen-centred Zn_4_O tetra­hedra as nodes but linked by different organic mol­ecules (Rosi *et al.*, 2003 ▸). As a control, we first successfully synthesized IRMOF-8, as already described, from H_2_NDC and Zn(NO_3_)_2_·6H_2_O in DEF using a common solvothermal route (Rowsell *et al.*, 2004 ▸). Under the same experimental conditions but in DEF containing *ca* 1.8% added formic acid, an unidentified crystalline powder was obtained, seemingly in a pure phase, that did not correspond to any known NDC-based MOF. However, in the presence of zinc(II) acetate as the metal source instead of zinc(II) nitrate, we successfully isolated a new 2D coordination network, [Zn(NDC)(DEF)]_*n*_ (**1**), identified by satisfactory elemental analysis and single-crystal X-ray diffraction.

## Structural commentary   

**Figure 1:**
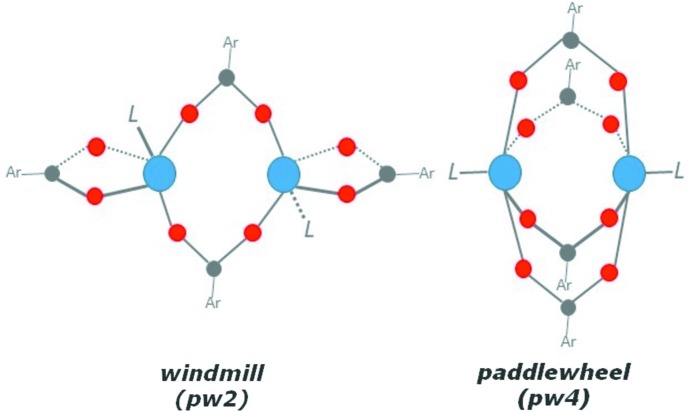
The structural model of the zinc windmill (or pw2) SBU found in MOF **1** (left) and of a typical zinc four-blade paddlewheel (pw4) cluster (right).

**Figure 2:**
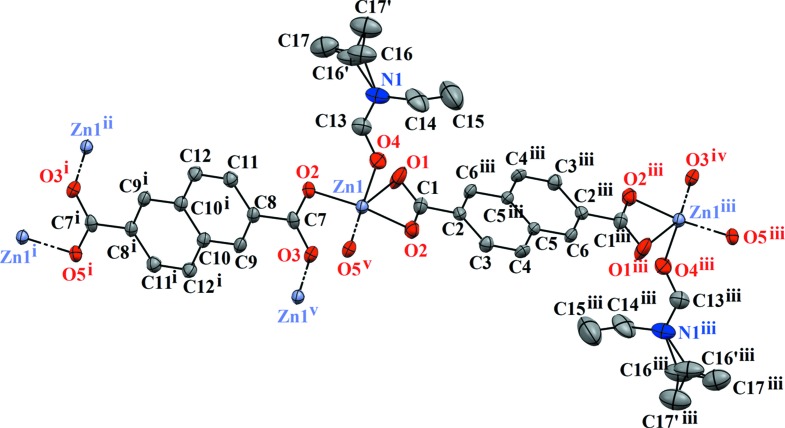
The mol­ecular structure of MOF **1**, with displacement ellipsoids drawn at the 50% probability level, showing the labelling sheme and the disordered ethyl group of DEF. [Symmetry codes: (i) −*x* + 1, −*y* − 1, −*z* + 1; (ii) *x* + 1, *y* − 1, *z*; (iii) −*x* − 1, −*y*, −*z* + 2; (iv) *x* − 1, *y*, *z* + 1; (v) −*x*, −*y*, −*z* + 1.]

Complex **1** crystallizes in the triclinic *P* 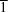 space group, with an asymmetric unit containing one Zn^2+^ cation, one fully deprotonated NDC^2−^ ligand and a Zn-coordinated DEF mol­ecule. Each Zn^II^ ion is penta­coordinated by five O atoms [Zn1—O1 = 2.543 (5) Å, Zn1—O2 = 1.949 (2) Å, Zn1—O3 = 2.026 (2) Å, Zn1—O4(DEF) = 1.979 (2) Å and Zn1—O5 = 1.980 (2) Å] from three individual NDC^2−^ anions and one DEF mol­ecule in a tetra­gonal pyramidal configuration. The SBU consists of doubly-bridged dinuclear units of Zn^II^ atoms in a ‘windmill’ fashion (Fig. 1 ▸), with a Zn⋯Zn distance of 3.652 (1) Å, where each pair of Zn atoms is linked by two NDC^2−^ anions and each Zn atom is linked by a further NDC^2−^ anion and a DEF mol­ecule (Fig. 2 ▸). The two carboxyl­ate groups of the same NDC^2−^ anion adopt either a μ_1_-η^1^:η^1^ (O1 and O2) or a μ_2_-η^1^:η^1^ (O3 and O5) coordination mode.
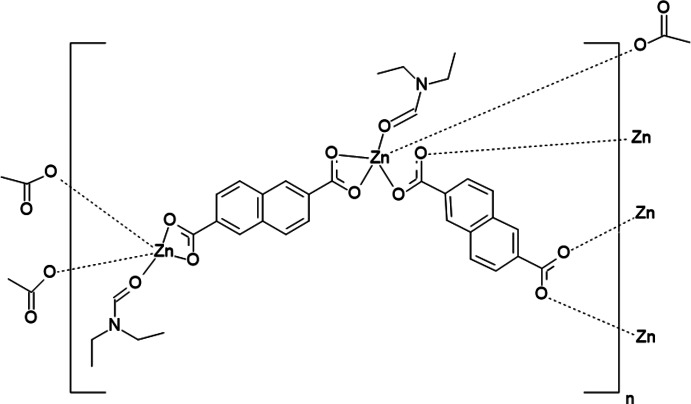


## Supra­molecular features   

**Figure 3:**
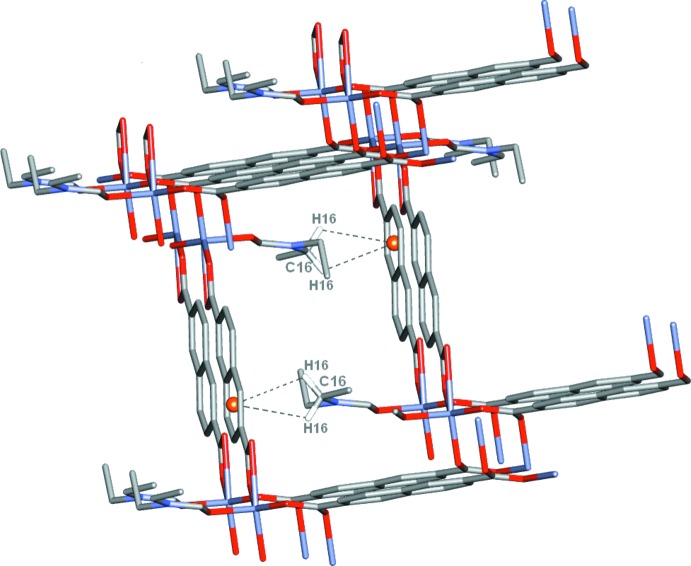
H_centroid_–π inter­action found in MOF **1** with DEF H atoms (H16) located near the centroid of the NDC^2−^ aromatic ring (all H atoms have been omitted for clarity, except for the DEF-H16 H atoms involved in the inter­actions).

**Figure 4:**
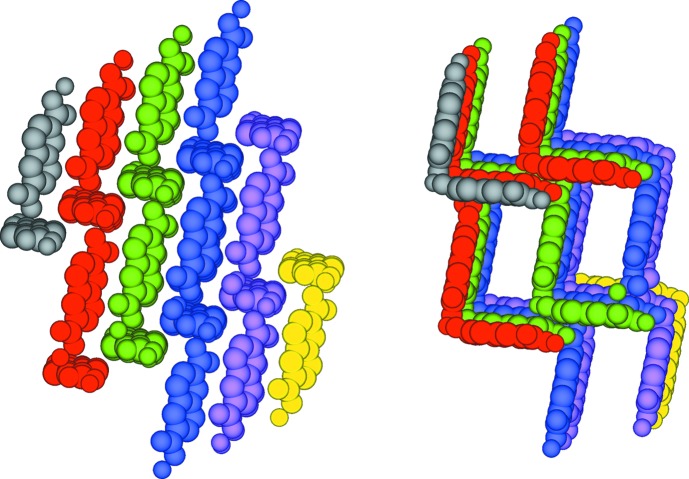
View of the two-dimensional layers in MOF **1** stacked in a self-locking fashion yielding the three-dimensional supra­molecular framework.

**Figure 5:**
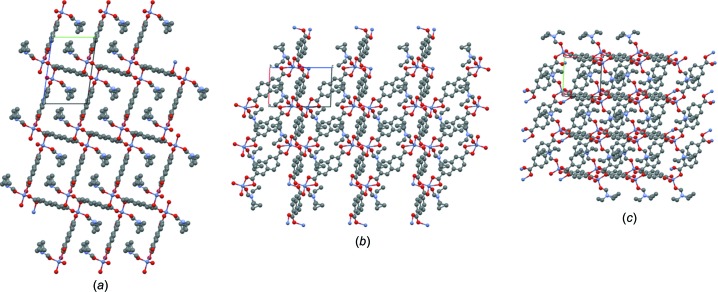
View of the two-dimensional stacked layers in MOF **1** along the crystallographic (*a*) *a*, (*b*) *b* and (*c*) *c* axes.

The structure of **1** shows a three-dimensional (3D) supramolecular framework built of zigzag-shaped two-dimensional (2D) stacked layers. Neighbouring 2D layers are inter­connected through nonclassical hydrogen-bonding inter­actions between carboxyl­ate O atoms (O1 and O3) and β-H atoms of NDC^2−^ ligands with COO⋯H—C_β_—NDC distances of 3.307 (4) (O1—C4) and 3.548 (4) Å (O3—C12). Other inter­actions contributing to the stability of the framework involve H_centroid_–π inter­actions of H16—C16 (DEF hydrogens) and the centroids [*Cg*1^iii^ is the centroid of the C2–C5/C5^v^/C6^v^ ring and *Cg*2^iv^ is the centroid of the C5/C6/C2^v^–C5^v^ ring; symmetry codes: (iii) *x* + 1, *y* + 1, *z*; (iv) −*x*, −*y* + 1, −*z* + 2; (v) −*x* − 1, −*y*, −*z* + 2] of the aromatic rings of the NDC^2−^ ligands, with *Cg*⋯H distances of 2.99 Å (Fig. 3 ▸ and Table 1 ▸). The layers are stacked in a self-locking fashion in a 3D supra­molecular framework (Fig. 4 ▸), which has open channels with dimensions of approximately 7.85 × 12.55 Å^2^ largely occupied by the Zn-coordinated DEF mol­ecules (Fig. 5 ▸). It is noteworthy that since **1** has been obtained in a DEF solution containing small amounts of formic acid, formate ligands are not present in the framework.

## Database survey   

Naphthalene di­carb­oxy­lic acid derivatives (H_2_NDCs), including 1,4-, 1,8- and 2,6-NDC, have been, due to their stability, richness in coordination modes and structural rigidity, widely used as organic mol­ecules in the synthesis of novel MOF structures with a variety of metal ions, such as Zn^II^, Cd^II^, Co^II^, Ni^II^, Mn^II^ or Ag^I^. Among all the 2,6-NDC/Zn-based MOFs, two are closely related to MOF **1**, *i.e.* a MOF of formula [Zn_2_(2,6-NDC)_2_(DMF)_2_]_*n*_ (Yang *et al.*, 2013 ▸), in which the two carboxyl­ate groups of all the NDC ligands have two different coordination modes (μ_1_-η^1^:η^1^ and μ_2_-η^1^:η^1^), and MOF-105 and its derivatives of generic formula [Zn_2_(2,6-NDC)_2_(DMF)_2_] (Eddaoudi *et al.*, 2002 ▸; Devi *et al.*, 2004 ▸; Shahangi Shirazi *et al.*, 2015 ▸; Yue *et al.*, 2015 ▸), in which all NDC-carboxyl­ates have a μ_2_-η^1^:η^1^ coordination mode, with a typical pw4 paddle-wheel structure motif, [*M*_2_(CO_2_)_4_]. For MOF **1**, the two carboxyl­ate groups of the same NDC^2−^ ligand adopt either a μ_1_-η^1^:η^1^ (O1 and O2) or a μ_2_-η^1^:η^1^ (O3 and O5) coordination mode, giving an uncommon pw2 paddle-wheel (‘windmill’) structural feature, [*M*_2_(CO_2_)_2_].

## Synthesis and crystallization   

**Figure 6:**
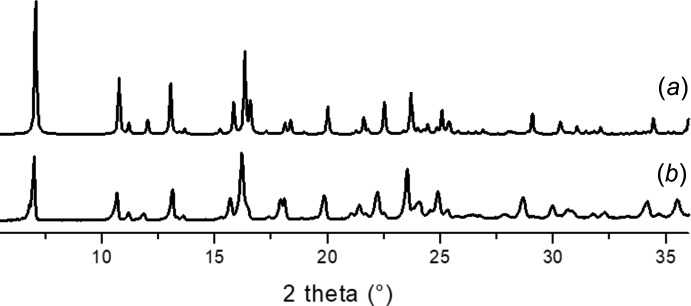
PXRD patterns (*a*) simulated from the single-crystal data of **1** and (*b*) measured from a sample of **1** prepared from 2,6-H_2_NDC and Zn(OAc)_2_ in DEF containing formic acid.

MOF **1** was synthesized from naphthalene-2,6-di­carb­oxy­lic acid and zinc(II) acetate. 2,6-H_2_NDC (87.3 mg, 0.4 mmol, 1.0 equiv.) and Zn(OAc)_2_·2H_2_O (224 mg, mol, 2.5 equiv.) were dissolved in DEF (10 ml) containing formic acid (185 µl, 12 equiv.) and sealed in a glass vial. The vial was heated in an oven to 110 °C for 17 h. After cooling to room temperature, the reaction was allowed to stand until colorless crystals suitable for X-ray diffraction formed. For further characterizations, the crystals were collected by filtration, washed with DEF several times, and dried at 373 K under vacuum. Ele­mental analysis (%) for C_17_H_17_NO_5_Zn based on the formula [Zn(NDC)(DEF)] found (calculated): C 53.00 (53.63), H 4.47 (4.50), N 3.39 (3.68), Zn 17.51 (17.17). FT–IR (cm^−1^): 2979, 2938, 1647, 1602, 1586, 1557, 1494, 1460, 1406, 1385, 1361, 1348. The identity of the as-synthesized bulk material was confirmed by com­paring the powder X-ray diffraction (PXRD) pattern with that simulated from the crystal structure (Fig. 6 ▸). After heating a sample of **1** at 463 K under vaccum for 8 h, coordinated DEF mol­ecules were eliminated, as evidenced by FT–IR (loss of bands at 2979, 2938 and 1647 cm^−1^). Elemental analysis (%) for C_12_H_6_O_4_Zn based on the formula [Zn(NDC)] found (calculated): C 48.85 (51.56), H 2.75 (2.16), N 0.22 (0.00), Zn 21.47 (23.39). It should be noted that after removal of DEF, MOF **1** lost its crystallinity, as evidenced by the PXRD pattern.

## Refinement   

The ethyl groups of DEF were disordered over two positions, for which the occupancies were refined, converging to 0.51 and 0.49. The SAME, DELU and SIMU restraints were applied to model the disorder (Sheldrick, 2008 ▸). All H atoms were fixed geometrically and treated as riding, with C—H = 0.95 (aromatic), 0.98 (CH_3_), 0.99 (CH_2_) or 1.0 Å (CH), with *U*_iso_(H) = 1.5*U*_eq_(C) for methyl H atoms or 1.2*U*_eq_(C) otherwise. Crystal data, data collection and structure refinement details are summarized in Table 2 ▸.

## Supplementary Material

Crystal structure: contains datablock(s) global. DOI: 10.1107/S2056989019014142/zl2761sup1.cif

CCDC references: 1959604, 1959604

Additional supporting information:  crystallographic information; 3D view; checkCIF report

## Figures and Tables

**Table 1:** Hydrogen-bond geometry (Å, °) *Cg*1 and *Cg*2 are the centroids of the C2–C5/C5^vii^/C6^vii^ and C5/C6/C2^vii^–C5^vii^ rings, respectively.

*D*—H⋯*A*	*D*—H	H⋯*A*	*D*⋯*A*	*D*—H⋯*A*
C4—H4⋯O1^iv^	0.95	2.39	3.307 (4)	161
C12—H12⋯O3^v^	0.95	2.63	3.548 (4)	156
C16—H16⋯*Cg*1^vi^	0.95	2.99	3.520 (17)	114
C16—H16⋯*Cg*2^vii^	0.95	2.99	3.520 (17)	114

Symmetry codes: (iv) 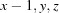; (v) 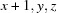; (vi) 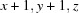; (vii) 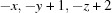.

**Table 2:** Experimental details

Crystal data	
Chemical formula	[Zn(C_12_H_6_O_4_)(C_15_H_11_NO)]
*M*_r_	380.68
Crystal system, space group	Triclinic, *P* 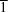
Temperature (K)	193
*a*, *b*, *c* (Å)	7.9134 (5), 8.3006 (5), 12.6413 (8)
α, β, γ (°)	97.873 (4), 91.620 (4), 91.991 (5)
*V* (Å^3^)	821.57 (9)
*Z*	2
Radiation type	Mo *K*α
μ (mm^−1^)	1.52
Crystal size (mm)	0.10 × 0.04 × 0.04

Data collection	
Diffractometer	Bruker SMART APEXII CCD area detector
Absorption correction	Multi-scan (*SADABS*; Bruker, 2008 ▸)
*T*_min_, *T*_max_	0.863, 0.942
No. of measured, independent and observed [*I* > 2σ(*I*)] reflections	13141, 3336, 2436
*R*_int_	0.075
(sin θ/λ)_max_ (Å^−1^)	0.625

Refinement	
*R*[*F* ^2^ > 2σ(*F* ^2^)], *wR*(*F* ^2^), *S*	0.042, 0.081, 1.00
No. of reflections	3336
No. of parameters	237
No. of restraints	41
H-atom treatment	H-atom parameters constrained
Δρ_max_, Δρ_min_ (e Å^−3^)	0.33, −0.37

Computer programs: *APEX2* (Bruker, 2008 ▸), *SAINT* (Bruker, 2008 ▸), *SHELXS97* (Sheldrick, 2008 ▸), *SHELXL2017* (Sheldrick, 2015 ▸), *SHELXTL* (Bruker, 2008 ▸) and *publCIF* (Westrip, 2010 ▸).

## supplementary crystallographic information

### Crystal data

**Table:**

[Zn(C_12_H_6_O_4_)(C_15_H_11_NO)]	*Z* = 2
*M**_r_* = 380.68	*F*(000) = 392
Triclinic, *P*1	*D*_x_ = 1.539 Mg m^−^^3^
Hall symbol: -P 1	Mo *K*α radiation, λ = 0.71073 Å
*a* = 7.9134 (5) Å	Cell parameters from 1701 reflections
*b* = 8.3006 (5) Å	θ = 2.5–21.5°
*c* = 12.6413 (8) Å	µ = 1.52 mm^−^^1^
α = 97.873 (4)°	*T* = 193 K
β = 91.620 (4)°	Block, colourless
γ = 91.991 (5)°	0.10 × 0.04 × 0.04 mm
*V* = 821.57 (9) Å^3^	

### Data collection

**Table:**

Bruker SMART APEXII CCD area detector diffractometer	3336 independent reflections
Radiation source: fine-focus selaed tube	2436 reflections with *I* > 2σ(*I*)
Detector resolution: 8.333 pixels mm^-1^	*R*_int_ = 0.075
phi and ω scans	θ_max_ = 26.4°, θ_min_ = 2.8°
Absorption correction: multi-scan (SADABS; Bruker, 2008)	*h* = −9→9
*T*_min_ = 0.863, *T*_max_ = 0.942	*k* = −10→10
13141 measured reflections	*l* = −15→15

### Refinement

**Table:**

Refinement on *F*^2^	Primary atom site location: structure-invariant direct methods
Least-squares matrix: full	Secondary atom site location: difference Fourier map
*R*[*F*^2^ > 2σ(*F*^2^)] = 0.042	Hydrogen site location: inferred from neighbouring sites
*wR*(*F*^2^) = 0.081	H-atom parameters constrained
*S* = 1.00	*w* = 1/[σ^2^(*F*_o_^2^) + (0.0318*P*)^2^] where *P* = (*F*_o_^2^ + 2*F*_c_^2^)/3
3336 reflections	(Δ/σ)_max_ = 0.001
237 parameters	Δρ_max_ = 0.33 e Å^−^^3^
41 restraints	Δρ_min_ = −0.37 e Å^−^^3^
0 constraints	

### Special details

**Table:**

Geometry. All esds (except the esd in the dihedral angle between two l.s. planes) are estimated using the full covariance matrix. The cell esds are taken into account individually in the estimation of esds in distances, angles and torsion angles; correlations between esds in cell parameters are only used when they are defined by crystal symmetry. An approximate (isotropic) treatment of cell esds is used for estimating esds involving l.s. planes.
Refinement. Refinement of F^2^ against ALL reflections. The weighted R-factor wR and goodness of fit S are based on F^2^, conventional R-factors R are based on F, with F set to zero for negative F^2^. The threshold expression of F^2^ > 2sigma(F^2^) is used only for calculating R-factors(gt) etc. and is not relevant to the choice of reflections for refinement. R-factors based on F^2^ are statistically about twice as large as those based on F, and R- factors based on ALL data will be even larger.

### Fractional atomic coordinates and isotropic or equivalent isotropic displacement parameters (Å^2^)

**Table:**

	*x*	*y*	*z*	*U*_iso_*/*U*_eq_	Occ. (<1)
Zn1	0.02528 (5)	0.13460 (5)	0.62683 (3)	0.02329 (12)	
O1	−0.0138 (3)	0.0539 (3)	0.8118 (2)	0.0495 (7)	
O2	−0.1959 (3)	0.1010 (3)	0.68613 (17)	0.0311 (5)	
O3	0.0472 (2)	−0.1661 (2)	0.52376 (16)	0.0249 (5)	
O4	0.1080 (3)	0.3581 (3)	0.68413 (19)	0.0376 (6)	
O5	0.2353 (2)	0.0121 (2)	0.60995 (16)	0.0267 (5)	
C1	−0.1604 (4)	0.0679 (4)	0.7800 (3)	0.0292 (8)	
C2	−0.3060 (4)	0.0478 (4)	0.8506 (2)	0.0229 (7)	
C3	−0.4729 (4)	0.0766 (4)	0.8157 (3)	0.0259 (7)	
H3	−0.491459	0.106685	0.746526	0.031*	
C4	−0.6065 (4)	0.0621 (4)	0.8792 (2)	0.0253 (7)	
H4	−0.716770	0.083839	0.854527	0.030*	
C5	−0.5832 (3)	0.0151 (3)	0.9818 (2)	0.0204 (7)	
C6	−0.7197 (4)	−0.0032 (4)	1.0501 (2)	0.0247 (7)	
H6	−0.831308	0.016042	1.026474	0.030*	
C7	0.1971 (4)	−0.1257 (4)	0.5574 (2)	0.0232 (7)	
C8	0.3342 (4)	−0.2464 (4)	0.5386 (2)	0.0231 (7)	
C9	0.2888 (4)	−0.4062 (4)	0.5012 (2)	0.0266 (7)	
H9	0.173329	−0.437095	0.484564	0.032*	
C10	0.4136 (4)	−0.5243 (4)	0.4875 (2)	0.0241 (7)	
C11	0.5046 (4)	−0.1977 (4)	0.5621 (3)	0.0281 (8)	
H11	0.534020	−0.086805	0.587033	0.034*	
C12	0.6283 (4)	−0.3091 (4)	0.5492 (3)	0.0285 (8)	
H12	0.743193	−0.274861	0.564835	0.034*	
C13	0.2535 (5)	0.4026 (4)	0.7188 (3)	0.0369 (9)	
H13	0.341309	0.327989	0.705702	0.044*	
C14	0.1574 (6)	0.6627 (5)	0.7929 (4)	0.0628 (13)	
H14A	0.078313	0.652571	0.729974	0.075*	
H14B	0.208053	0.774681	0.803717	0.075*	
C15	0.0611 (7)	0.6341 (6)	0.8898 (4)	0.0970 (18)	
H15A	0.013080	0.522378	0.879973	0.145*	
H15B	−0.030364	0.710761	0.899858	0.145*	
H15C	0.137829	0.650364	0.952935	0.145*	
N1	0.2922 (4)	0.5454 (4)	0.7719 (2)	0.0448 (8)	
C16	0.4576 (17)	0.6215 (19)	0.815 (2)	0.061 (4)	0.516 (8)
H16A	0.487379	0.713730	0.775639	0.074*	0.516 (8)
H16B	0.447852	0.665014	0.890959	0.074*	0.516 (8)
C17	0.5911 (12)	0.5053 (11)	0.8035 (8)	0.065 (3)	0.516 (8)
H17A	0.563272	0.415235	0.843378	0.098*	0.516 (8)
H17B	0.698301	0.559271	0.831750	0.098*	0.516 (8)
H17C	0.601884	0.462995	0.727791	0.098*	0.516 (8)
C16'	0.4774 (18)	0.575 (2)	0.8054 (19)	0.062 (4)	0.484 (8)
H16C	0.544874	0.496333	0.760003	0.074*	0.484 (8)
H16D	0.513676	0.685674	0.792220	0.074*	0.484 (8)
C17'	0.5149 (13)	0.5600 (11)	0.9178 (7)	0.074 (3)	0.484 (8)
H17D	0.426986	0.611745	0.962205	0.110*	0.484 (8)
H17E	0.625043	0.613689	0.939854	0.110*	0.484 (8)
H17F	0.517878	0.444637	0.926528	0.110*	0.484 (8)

### Atomic displacement parameters (Å^2^)

**Table:**

	*U*^11^	*U*^22^	*U*^33^	*U*^12^	*U*^13^	*U*^23^
Zn1	0.0191 (2)	0.0234 (2)	0.0272 (2)	0.00401 (14)	0.00315 (15)	0.00116 (15)
O1	0.0203 (14)	0.086 (2)	0.0477 (17)	0.0087 (13)	0.0085 (12)	0.0236 (15)
O2	0.0277 (13)	0.0404 (14)	0.0263 (13)	0.0025 (10)	0.0067 (11)	0.0068 (11)
O3	0.0184 (12)	0.0277 (12)	0.0302 (13)	0.0040 (9)	0.0037 (10)	0.0087 (10)
O4	0.0358 (15)	0.0268 (13)	0.0474 (16)	0.0019 (10)	0.0016 (12)	−0.0052 (11)
O5	0.0258 (12)	0.0249 (13)	0.0286 (13)	0.0078 (9)	0.0039 (10)	−0.0010 (10)
C1	0.0237 (19)	0.0315 (19)	0.033 (2)	0.0043 (14)	0.0064 (16)	0.0037 (16)
C2	0.0208 (17)	0.0233 (17)	0.0232 (18)	0.0008 (13)	0.0028 (14)	−0.0022 (14)
C3	0.0250 (18)	0.0267 (18)	0.0270 (19)	0.0046 (14)	0.0008 (15)	0.0064 (15)
C4	0.0172 (17)	0.0308 (19)	0.0277 (19)	0.0021 (13)	−0.0044 (14)	0.0042 (15)
C5	0.0170 (16)	0.0211 (16)	0.0222 (17)	0.0014 (12)	−0.0003 (13)	0.0003 (13)
C6	0.0143 (16)	0.0288 (18)	0.0308 (19)	0.0031 (13)	−0.0004 (14)	0.0027 (15)
C7	0.0287 (19)	0.0256 (18)	0.0176 (17)	0.0068 (14)	0.0075 (14)	0.0079 (14)
C8	0.0220 (17)	0.0257 (18)	0.0224 (18)	0.0067 (13)	0.0039 (14)	0.0046 (14)
C9	0.0188 (17)	0.0334 (19)	0.0284 (19)	0.0058 (14)	0.0032 (14)	0.0050 (15)
C10	0.0215 (17)	0.0276 (18)	0.0241 (17)	0.0035 (13)	0.0036 (14)	0.0049 (14)
C11	0.0274 (19)	0.0260 (19)	0.031 (2)	0.0038 (14)	0.0026 (15)	0.0018 (15)
C12	0.0233 (18)	0.0271 (18)	0.034 (2)	−0.0003 (14)	0.0039 (15)	0.0011 (15)
C13	0.042 (2)	0.032 (2)	0.037 (2)	0.0006 (16)	−0.0029 (18)	0.0063 (17)
C14	0.089 (4)	0.027 (2)	0.067 (3)	−0.006 (2)	0.010 (3)	−0.012 (2)
C15	0.111 (5)	0.084 (4)	0.087 (4)	−0.011 (3)	0.037 (4)	−0.023 (3)
N1	0.057 (2)	0.0383 (19)	0.0367 (19)	−0.0130 (16)	−0.0097 (16)	0.0030 (15)
C16	0.077 (6)	0.051 (8)	0.053 (6)	−0.018 (5)	−0.018 (5)	0.006 (6)
C17	0.063 (6)	0.069 (6)	0.066 (6)	−0.014 (4)	−0.011 (5)	0.022 (5)
C16'	0.076 (6)	0.052 (9)	0.055 (6)	−0.028 (6)	−0.023 (6)	0.013 (7)
C17'	0.100 (7)	0.061 (6)	0.059 (6)	−0.021 (5)	−0.016 (5)	0.015 (5)

### Geometric parameters (Å, º)

**Table:**

Zn1—O2	1.949 (2)	C11—C12	1.368 (4)
Zn1—O4	1.979 (2)	C11—H11	0.9500
Zn1—O5	1.980 (2)	C12—H12	0.9500
Zn1—O3^i^	2.026 (2)	C13—N1	1.302 (4)
Zn1—C1	2.571 (3)	C13—H13	0.9500
O1—C1	1.231 (4)	C14—N1	1.474 (5)
O2—C1	1.280 (4)	C14—C15	1.503 (6)
O3—C7	1.267 (3)	C14—H14A	0.9900
O4—C13	1.246 (4)	C14—H14B	0.9900
O5—C7	1.264 (4)	C15—H15A	0.9800
C1—C2	1.496 (4)	C15—H15B	0.9800
C2—C6^ii^	1.368 (4)	C15—H15C	0.9800
C2—C3	1.419 (4)	N1—C16	1.488 (11)
C3—C4	1.358 (4)	N1—C16'	1.517 (11)
C3—H3	0.9500	C16—C17	1.452 (17)
C4—C5	1.413 (4)	C16—H16A	0.9900
C4—H4	0.9500	C16—H16B	0.9900
C5—C6	1.420 (4)	C17—H17A	0.9800
C5—C5^ii^	1.424 (5)	C17—H17B	0.9800
C6—H6	0.9500	C17—H17C	0.9800
C7—C8	1.504 (4)	C16'—C17'	1.47 (2)
C8—C9	1.378 (4)	C16'—H16C	0.9900
C8—C11	1.406 (4)	C16'—H16D	0.9900
C9—C10	1.413 (4)	C17'—H17D	0.9800
C9—H9	0.9500	C17'—H17E	0.9800
C10—C12^iii^	1.422 (4)	C17'—H17F	0.9800
C10—C10^iii^	1.426 (6)		
			
O2—Zn1—O4	107.22 (9)	C8—C11—H11	119.8
O2—Zn1—O5	136.08 (9)	C11—C12—C10^iii^	120.5 (3)
O4—Zn1—O5	103.46 (9)	C11—C12—H12	119.7
O2—Zn1—O3^i^	99.73 (8)	C10^iii^—C12—H12	119.7
O4—Zn1—O3^i^	100.55 (9)	O4—C13—N1	123.8 (3)
O5—Zn1—O3^i^	104.71 (8)	O4—C13—H13	118.1
O2—Zn1—C1	28.93 (9)	N1—C13—H13	118.1
O4—Zn1—C1	100.44 (10)	N1—C14—C15	111.5 (4)
O5—Zn1—C1	115.09 (9)	N1—C14—H14A	109.3
O3^i^—Zn1—C1	128.55 (9)	C15—C14—H14A	109.3
C1—O2—Zn1	103.60 (19)	N1—C14—H14B	109.3
C7—O3—Zn1^i^	119.47 (19)	C15—C14—H14B	109.3
C13—O4—Zn1	127.3 (2)	H14A—C14—H14B	108.0
C7—O5—Zn1	107.70 (19)	C14—C15—H15A	109.5
O1—C1—O2	122.1 (3)	C14—C15—H15B	109.5
O1—C1—C2	121.0 (3)	H15A—C15—H15B	109.5
O2—C1—C2	116.8 (3)	C14—C15—H15C	109.5
O1—C1—Zn1	74.8 (2)	H15A—C15—H15C	109.5
O2—C1—Zn1	47.47 (15)	H15B—C15—H15C	109.5
C2—C1—Zn1	163.8 (2)	C13—N1—C14	118.8 (3)
C6^ii^—C2—C3	119.1 (3)	C13—N1—C16	131.2 (8)
C6^ii^—C2—C1	120.6 (3)	C14—N1—C16	110.0 (8)
C3—C2—C1	120.3 (3)	C13—N1—C16'	115.1 (9)
C4—C3—C2	121.2 (3)	C14—N1—C16'	126.1 (9)
C4—C3—H3	119.4	C17—C16—N1	111.5 (12)
C2—C3—H3	119.4	C17—C16—H16A	109.3
C3—C4—C5	120.7 (3)	N1—C16—H16A	109.3
C3—C4—H4	119.7	C17—C16—H16B	109.3
C5—C4—H4	119.7	N1—C16—H16B	109.3
C4—C5—C6	122.4 (3)	H16A—C16—H16B	108.0
C4—C5—C5^ii^	119.0 (3)	C16—C17—H17A	109.5
C6—C5—C5^ii^	118.6 (3)	C16—C17—H17B	109.5
C2^ii^—C6—C5	121.4 (3)	H17A—C17—H17B	109.5
C2^ii^—C6—H6	119.3	C16—C17—H17C	109.5
C5—C6—H6	119.3	H17A—C17—H17C	109.5
O5—C7—O3	122.1 (3)	H17B—C17—H17C	109.5
O5—C7—C8	118.2 (3)	C17'—C16'—N1	114.1 (15)
O3—C7—C8	119.6 (3)	C17'—C16'—H16C	108.7
C9—C8—C11	120.8 (3)	N1—C16'—H16C	108.7
C9—C8—C7	118.7 (3)	C17'—C16'—H16D	108.7
C11—C8—C7	120.5 (3)	N1—C16'—H16D	108.7
C8—C9—C10	120.1 (3)	H16C—C16'—H16D	107.6
C8—C9—H9	119.9	C16'—C17'—H17D	109.5
C10—C9—H9	119.9	C16'—C17'—H17E	109.5
C9—C10—C12^iii^	121.8 (3)	H17D—C17'—H17E	109.5
C9—C10—C10^iii^	119.2 (4)	C16'—C17'—H17F	109.5
C12^iii^—C10—C10^iii^	118.9 (3)	H17D—C17'—H17F	109.5
C12—C11—C8	120.4 (3)	H17E—C17'—H17F	109.5
C12—C11—H11	119.8		

Symmetry codes: (i) −*x*, −*y*, −*z*+1; (ii) −*x*−1, −*y*, −*z*+2; (iii) −*x*+1, −*y*−1, −*z*+1.

### Hydrogen-bond geometry (Å, º)

*Cg*1 and *Cg*2 are the centroids of the C2–C5/C5^ii^/C6^ii^ and C5/C6/C2^ii^–C5ii rings, respectively. [Symmetry code: (ii) -x-1, -y, -z+2.]

**Table:**

*D*—H···*A*	*D*—H	H···*A*	*D*···*A*	*D*—H···*A*
C4—H4···O1^iv^	0.95	2.39	3.307 (4)	161
C12—H12···O3^v^	0.95	2.63	3.548 (4)	156
C16—H16···*Cg*1^vi^	0.95	2.99	3.520 (17)	114
C16—H16···*Cg*2^vii^	0.95	2.99	3.520 (17)	114

Symmetry codes: (iv) *x*−1, *y*, *z*; (v) *x*+1, *y*, *z*; (vi) *x*+1, *y*+1, *z*; (vii) −*x*, −*y*+1, −*z*+2.
